# The Influence of Cations and Host–Guest Interactions on Alginate Gels Properties

**DOI:** 10.3390/gels12030217

**Published:** 2026-03-06

**Authors:** Gabriela Ioniță, Carmen Mihaela Topală, Elena-Erika Antonia, Mihaela Lavinia Ciutu, Alexandru Gabriel Bucur, Nusa Elena Hristea, Rodica Baratoiu, Ludmila Aricov, Anca Ruxandra Leonties, Carla-Cezarina Pădurețu

**Affiliations:** 1 Institute of Physical Chemistry–Ilie Murgulescu, Romanian Academy, 202 Splaiul Independentei, 060021 Bucharest, Romania; ige@icf.ro (G.I.); abucur@icf.ro (A.G.B.); laricov@icf.ro (L.A.); aleonties@icf.ro (A.R.L.); 2 Faculty of Sciences, Physical Education and Computer Science, The National University of Science and Technology Politehnica Bucharest, Pitesti University Centre, 1 Targu din Vale Street, 110040 Pitesti, Romania; carmen.topala@upb.ro

**Keywords:** alginate, hydrogels, EPR spectroscopy, cyclodextrin

## Abstract

Ionotropic alginate-based hydrogelation by divalent metal interaction has been employed to study the effect that different types of ions might have on gel formation. In this regard, EPR and IR spectroscopies, as well as rheology techniques, have been used to evaluate the influence of divalent cations on gel formation, and at the same time to assess host–guest interactions. Alginate was functionalized with TEMPO moieties; therefore, TEMPO-alginate system was taken as a reference. The novelty of this study consists of using a mixture of adamantyl-TEMPO-functionalized alginate and β-cyclodextrin linked through 1,3-diaminopropane to assess the host–guest interactions in functionalized gels. The properties of divalent cations considered in this study (Ba^2+^, Ca^2+^, Sr^2+^, Zn^2+^) were proven by changes in spectral parameters of paramagnetic moieties, while the viscoelastic moduli as functions of shear strain and frequency were evaluated through rheology measurements. Overall, the information obtained from these investigations has shown that the properties of the alginate gels are influenced both by the type of divalent cation used for complexation and by the host–guest interactions. The results show that the type of the cation significantly affects gel strength; therefore, Ba^2+^ forms the strongest gel, while Zn^2+^ the least resistant. Additionally, a high immobilization of the spin-labeled probes has been obtained by the addition of tosylated β-cyclodextrin in the alginate gel network containing Ba^2+^ ions.

## 1. Introduction

**Figure 1:**
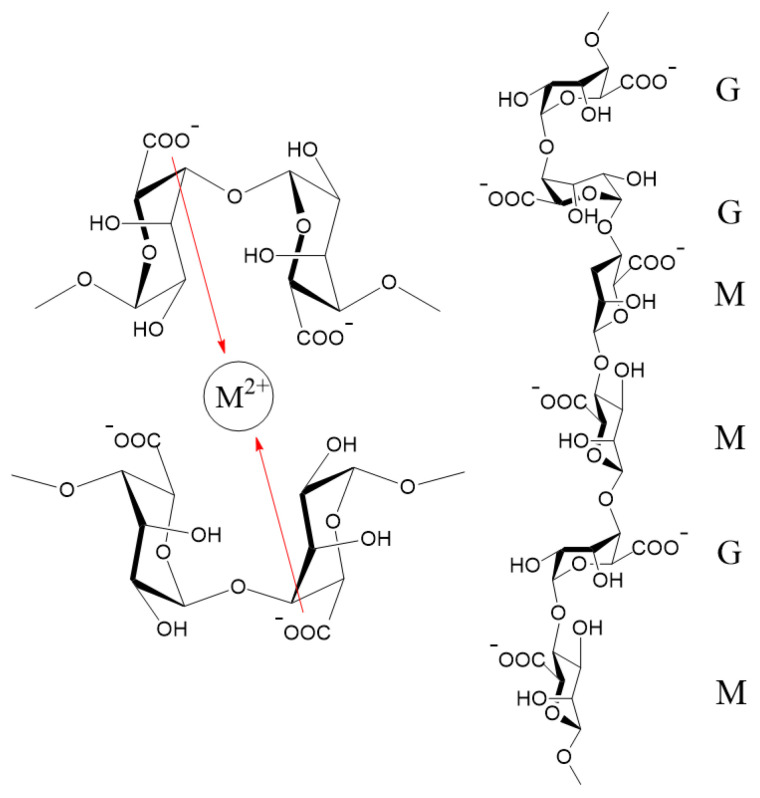
Possible connections in an “egg-box” model of an M^2+^ ion in a divalent alginate gel with a predominant cross-link between carboxylate groups (**left**), and structural characteristics of chain conformation of alginate (**right**). Adapted from Refs. [3,13].

Alginate is a naturally occurring biopolymer mainly found in the cell walls of algae and bacteria. The structure of alginate could be modified as such one could obtain hydrogels, nanoparticles, films, and membranes due to good mechanical properties of the obtained materials such as high viscoelasticity [1]. Alginate is a linear copolymer of α-L-mannuronic acid (M) and β-D-guluronic acid (G), exhibiting a strong affinity for divalent ions which contribute to cross-linking alginate polymers into hydrogel networks through G-blocks or alternating M- and G-blocks, also known as MG-blocks [2]. After entering the alginate matrix, divalent ions begin coordinating with the negatively charged carboxylate groups (–COO^−^) of guluronic acid (G) residues. In these regions, G-blocks take an ordered conformation that enables cooperative chelation: pairs of guluronic acid chains align such that their carboxylate and hydroxyl groups form well-defined cavities capable of hosting metal ions [3]. This arrangement is explained through the so-called “egg-box” model, where guluronic acid blocks, which serve as the “boxes”, cooperate with counterions, known as the “eggs”, to stabilize junction zones [4] as shown in Figure 1. Divalent ions migrate through the hydrated polymer matrix until they reach the available binding sites on the alginate chains. Different factors such as the ionic radius and charge density of the metal ions may affect the binding strength and cross-linking density [5]. For example, the binding of divalent cations to G-blocks is highly selective. The affinity increases in the following order: Zn^2+^ < Ca^2+^ < Sr^2+^ < Ba^2+^ [6]. The cross-linking of M-blocks with barium is more effective than calcium and strontium ions, though barium could cross-link the MG-blocks less effectively [7]. Additionally, the diffusion rates vary among ions as follows: Ca^2+^ and Sr^2+^ penetrate relatively quickly, while Ba^2+^ moves more slowly due to its larger hydrated radius [8,9]. Although Ca^2+^ and Ba^2+^ are the most commonly investigated cross-linkers, examining a wider range of divalent or mixed-metal ions demonstrates that the choice of cation can significantly impact gel network structure, porosity, and dynamic behavior, due to variations in ionic size, coordination geometry, and binding affinity [10,11,12]. Calcium ions (Ca^2+^) are the most widely used cross-linkers for alginate, possessing a binding affinity well-matched to the polymer [13]. Calcium typically coordinates with pairs of G- and MG-blocks to create networks that are moderately strong but flexible at the same time. Barium ions (Ba^2+^), by contrast, generate much stronger cross-links due to their larger ionic radius and higher affinity for G- and M-blocks, producing dense, highly stable gels with reduced permeability. Strontium ions (Sr^2+^) were found to bind to G-blocks solely. Additionally, strontium has the ionic size between Ca^2+^ and Ba^2+^, producing gels with intermediate rigidity; it has stiffer and higher stability than Ca-alginate, but less rigidity than Ba-alginate [2,9,14]. Strontium-alginate allows for the transport of oxygen and nutrients to be tuned through polymer composition and cross-linker concentration [5,15]. Zinc ions (Zn^2+^) follow a more complex coordination mechanism since they bind not only to G-blocks but also to M-blocks. As a result, Zn-alginate gels tend to be stiff but brittle, with a high degree of flexibility in their chemical responsiveness [9]. Zinc alginate typically develops smaller, more irregular pore structures, a feature that supports controlled drug release as well as metal ion adsorption in environmental remediation systems [16,17].

The internal structure of alginate gels strongly contributes to changes in the mesh size, porosity, and cross-link density which determine its stiffness, diffusional properties, swelling behavior, and stability under different conditions [18]. The physical–chemical properties of alginate gels determine their applications as encapsulating and delivery systems for drugs, proteins, cells, etc., or as components in food packaging [15,19,20].

Due to the hydrophilic character of alginate gels, their use as delivery drug agents is limited to water soluble compounds that can be uploaded. To extend the applicability of these types of gels as drug carriers for hydrophobic compounds and to gain better control over the loading and release of therapeutic agents from hydrogels, it is possible to functionalize the alginate chains with cyclodextrins [21].

In previous studies, the formation of alginate gels and the effect of host–guest interactions occurring in functionalized alginate gels using Ca^2+^ as gelation cation by rheology, EPR, and IR spectroscopies have been investigated [22,23].

The present study is focused on the effect of the binding cations in similar systems. Therefore, we have used in this study the alginate functionalized with TEMPO moieties, alginate functionalized with adamantyl and TEMPO moieties, and alginate functionalized with β-cyclodextrin (β-CD) linked to a 1,3-diaminopropane chain. The corresponding alginate gels were formed in the presence of the following cations: Ca^2+^, Sr^2+^, Ba^2+^, and Zn^2+^, using EPR and IR spectroscopies and rheological measurements.

## 2. Results and Discussion

The alginate gels obtained by the complexation of functionalized alginates with divalent cations were characterized by IR spectroscopy and rheological measurements to obtain information at a global level, and EPR spectroscopy to obtain information at a local level around paramagnetic moieties that can provide an overall characterization of these materials.

### 2.1. Infrared Spectroscopy

**Figure 2:**
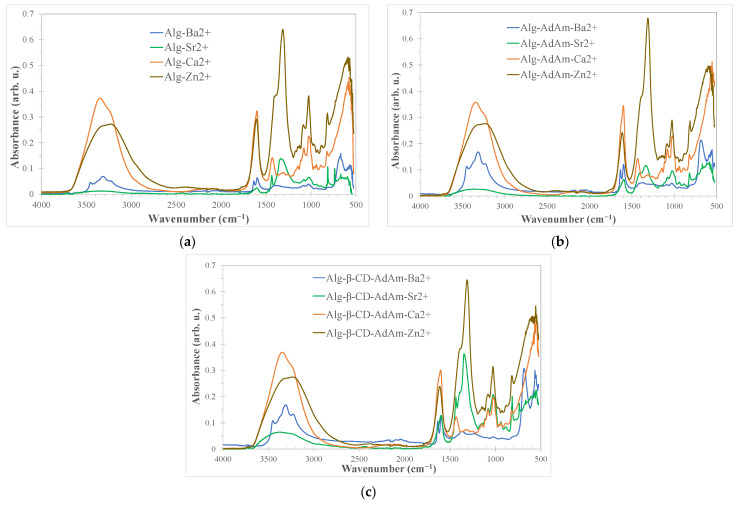
Infrared spectra of spin-labeled alginate: (**a**) in the presence of divalent ions, (**b**) in the presence of divalent ions and adamantyl, (**c**) in the presence of cyclodextrin.

The infrared spectra of functionalized alginate gels presented the bands characteristic for vibrational modes associated with the structural groups in the polysaccharide units and of the moieties attached to the chains. The IR spectra of dried alginate gels are plotted in Figure 2, and the assignments of main vibration bands are presented in Table S1 (Supplementary Materials). The gels were dried in order to diminish the signal of water that can cover the infrared bands of the chain, as water can interfere with the infrared spectra in the 3600–3000 cm^−1^ region where stretching vibrations of ν(OH) groups are usually shown, as well as in the fingerprint region, masking coordination-related band shifts. Drying allows a spectral observation of the band shifts associated with coordination (e.g., C=O, COO^−^, or other ligand-related vibrations) to be obtained. The coordination bonds between metal ions and polymer ligands were already present in the hydrogel network and were responsible for gel formation. Although drying may alter the structural arrangement or hydration sphere, it did not fundamentally change the chemical identity of the coordination interactions.

The IR spectral bands observed between 3600 and 3000 cm^−1^ could also be assigned to intramolecular and intermolecular hydrogen bonding from OH groups. It can be noted that the nature of the cation influences the fingerprint of these vibration bands, as they can coordinate differently with the polysaccharide chain. In the case of the alginate gels formed in the presence of Ba^2+^, the presence of three bands instead of one broad band in the region 3600–3000 cm^−1^ can be observed. The IR bands attributed to the asymmetric, ν_a_ (COO^−^), and symmetric, ν_s_ (COO^−^), stretching vibrations are found in the region 1700–1400 cm^−1^. The ν_a_ (COO^−^) vibration occur around 1600 cm^−1^ and, from the data presented in Table S1, it was not very sensitive to the type of cation. In the case of Ba^2+^, the presence of two split bands around 1635 and 1600 cm^−1^ is observed, while for the other cations these bands appeared to be formed as a single band. The bands corresponding to symmetric vibrations of the COO^−^ group ν_s_ (COO^−^) were shifted when using different ions. For Ca^2+^ and Sr^2+^, the vibration bands ν_s_ (COO^−^) appeared around 1430 cm^−1^, while Zn^2+^ and Ba^2+^ were shifted to lower wavenumbers around 1400 cm^−1^ in the case of non-functionalized alginate. For functionalized alginate gels, either with adamanatyl group or adamantyl and cyclodextrin units formed by cross-linking with Ba^2+^, a more significant shift in the vibration bands ν_s_ (COO^−^) positioned toward 1363 cm^−1^ was identified. The CO stretching vibrations observed around 1025 cm^−1^ were not sensitive to either the cation type or the covalent functionalization of the alginate chain.

In general, for all the gels obtained, it was observed that for zinc nitrate and strontium nitrate a specific peak assigned to stretching vibrations of nitrate ions (NO^3−^) [24] appears at 1309 and 1336 cm^−1^, respectively. However, since nitrate was not directly involved in complexation reactions, the role of this anion could be considered as negligible.

### 2.2. Rheology

**Figure 3:**
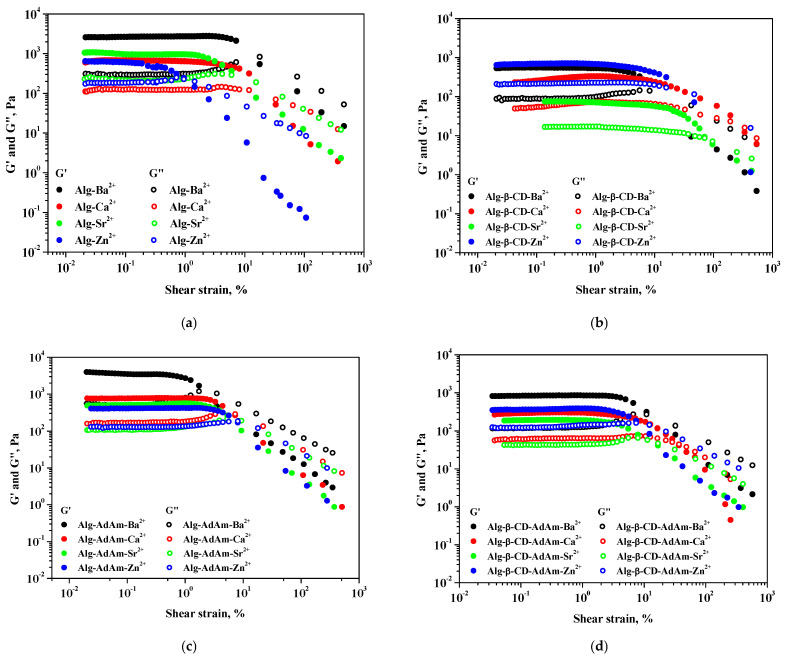
Amplitude sweep measurements (G′ and G″ as a function of shear strain) for: (**a**) Alg, (**b**) Alg-β-CD, (**c**) Alg-AdAm, and (**d**) Alg-β-CD-AdAm hydrogels cross-linked with different divalent ions.

**Figure 4:**
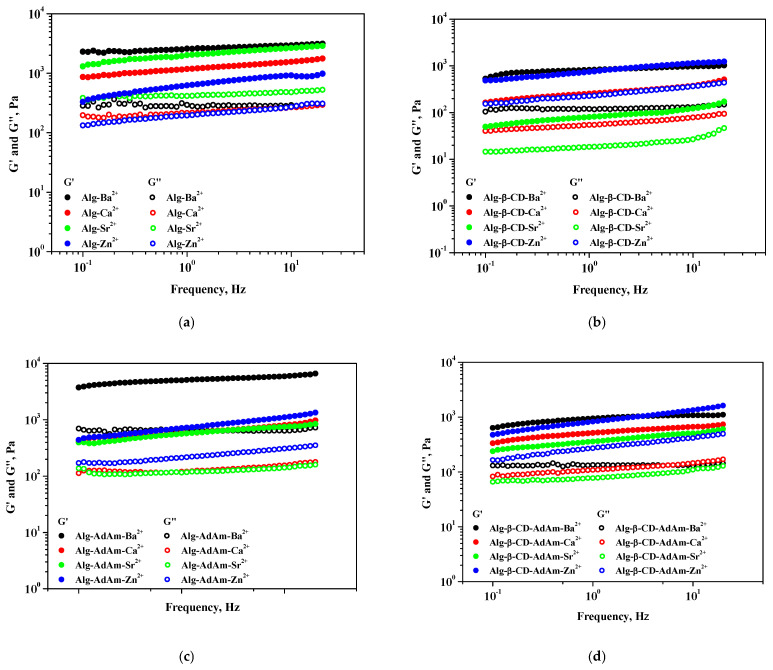
Frequency sweep measurements (G′ and G″ as a function of frequency) for (**a**) Alg, (**b**) Alg-β-CD, (**c**) Alg-AdAm, and (**d**) Alg-β-CD-AdAm hydrogels cross-linked with different divalent ions.

The impact of divalent ions such as Ca^2+^, Ba^2+^, Sr^2+^, and Zn^2+^ on alginate-based systems was examined through rheological analysis. Figure 3 and Figure 4 present measurements of viscoelastic moduli as functions of shear strain and frequency, respectively, for Alg, Alg-β-CD, Alg-AdAm, and Alg-β-CD-AdAm hydrogels cross-linked with different divalent ions. The two rheological moduli are the storage/elastic modulus (G′) and the loss/viscous modulus (G″). Furthermore, Table 1 displays the values of the two viscoelastic moduli at 0.5% shear strain for the alginate-based systems in the presence of divalent ions.

All examined alginate-based hydrogels demonstrated a primarily elastic behavior, as indicated by G′ > G″, in the linear viscoelastic (LVE) region. Amplitude sweep experiments demonstrated consistent LVE plateaus across all systems, succeeded by a gradual decline in G′ at higher shear strain values, suggesting a deformation of the ionic cross-linked networks. Measurements conducted through frequency sweeps (Figure 4) in the LVE region revealed a relatively weak frequency dependence of G′, which supported that the hydrogel networks were primarily governed by ionic and supramolecular interactions (particularly in the Alg-β-CD-AdAm system).

Viscoelastic behavior presented a significant dependence on the polymeric system as well as the nature of the ions. Among all the investigated systems, pure Alg hydrogels exhibited the highest G′, especially when cross-linked with Ba^2+^, reaching a G′ value of 2727 Pa at 0.5% strain. This behavior was characteristic of a dense ionic network formed by the classical “egg-box” model, where Ba^2+^ ions can efficiently coordinate the guluronate-rich regions of the alginate chains [19]. Substitution with Ca^2+^ and Sr^2+^ resulted in lower G′ values, reflecting reduced ionic binding strength and cross-link density, while Zn^2+^ produced comparatively softer gels, suggesting less efficient alginate coordination.

The functionalization of alginate with β-cyclodextrin (Alg-β-CD) led to a significant decrease in the elastic modulus for the majority of cross-linking ions. The G′ value decreased to 548 Pa for Ba^2+^ and further declined to 73.7 Pa for Sr^2+^. Also, by complexation of β-CD with Sr^2+^ ions, the alginate networks were weaker compared to the other ions. The observed decrease suggested that the β-CD groups disrupted the dense packing of the alginate chain, hindering the development of extensive ionic junction regions. Zn^2+^-cross-linked Alg-β-CD gels demonstrated a higher G′ (708 Pa), indicating that Zn^2+^ might participate in additional coordination or specific interactions with the β-CD units, which could partially compensate for the diminished alginate–alginate interactions. It was observed that the G′ value for Sr^2+^ in Alg-β-CD decreased sharply (73.7 Pa) compared to the G′ value for Ca^2+^ (319.4 Pa), which could be explained by the cation affinity, since Ca^2+^ ions typically coordinates with pairs of G- and MG-blocks to create cross-linked networks, while Sr^2+^ ions were found to solely bind to G-blocks. The presence of strontium may cause differences in the structural arrangement of alginate, which may have led to obtaining a different G′ value in the presence of β-CD.

In contrast, adamantyl-modified alginate (Alg-AdAm) demonstrated improved viscoelastic properties, especially in the presence of Ba^2+^, with the highest recorded G′ value of 3270 Pa among all systems. The presence of hydrophobic adamantyl units likely increased chain rigidity and facilitated more efficient stress transfer through the network while having a low impact on ionic cross-linking. Compared to Alg-β-CD, Alg-AdAm demonstrated consistently higher G′ values for all cross-linking ions, suggesting that adamantane functionalization improved the mechanical strength of Alg.

Intermediate viscoelastic behavior was observed in the Alg-β-CD-AdAm mixtures. This behavior reflected the coexistence of ionic cross-links and supramolecular host–guest interactions between β-CD cavities and AdAm units. These systems exhibited G′ values of 850 Pa (Ba^2+^), 302 Pa (Ca^2+^), and 379 Pa (Zn^2+^), indicating a balance between the weaker viscoelastic response of Alg-β-CD and the enhanced mechanical strength of Alg-AdAm. Therefore, the host–guest β-CD/AdAm interactions introduced additional physical cross-links that partially strengthened the network compared to Alg-β-CD alone while remaining weaker than the Alg-AdAm system. In comparison to Alg-β-CD alone, the mixture exhibited greater G′ values, thereby confirming the strengthening effect of AdAm inclusion, while still maintaining a softer consistency than Alg-AdAm systems due to the steric constraints imposed by β-CD.

The type of cross-linking ion significantly influenced the stiffness of the hydrogel across all systems. Ba^2+^ generally produced the most elastic and stable gel networks, followed by Ca^2+^ and Sr^2+^, whereas Zn^2+^ produced softer gels. The loss modulus values were consistently lower than the storage modulus across all samples, thereby confirming the elastic dominance of the hydrogels. The results indicated that the rheological properties of alginate-based hydrogels can be effectively adjusted through polymer functionalization, supramolecular host–guest interactions, and selective ionic cross-linking. Ba^2+^ enhanced network rigidity via strong ionic junctions, while the addition of β-CD and AdAm facilitated the modulation of elasticity and damping behavior through host–guest interactions. This approach could offer a flexible strategy for the design of adaptive and multifunctional hydrogel systems.

The ionic cross-linking of the Alg-based system with MgCl_2_ was further examined. In contrast to other divalent cations, Mg^2+^ did not induce gel formation. In this context, the shear viscosity value was assessed at a constant shear rate of 0.1 s^−1^. The obtained values were 9.8 × 10^−2^ Pa s for Alg-Mg^2+^, 5.0 × 10^−2^ Pa s for Alg-β-CD-Mg^2+^, and 1.1 × 10^−1^ Pa s for Alg-AdAm-Mg^2+^. The low viscosities suggested that Mg^2+^ did not facilitate gelation. The presence of β-CD slightly decreased the viscosity, suggesting a potential disruption of polymer chain interactions. The presence of AdAm resulted in an insignificant increase in viscosity, indicating weak interactions, which slightly restricted the mobility of the polymer chain. The findings demonstrated that, despite Alg modifications with β-CD or AdAm, the Mg^2+^ systems continued to exhibit liquid-like properties. This behavior was due to the small ionic radius and strong hydration shell of Mg^2+^, which restricted its capacity to effectively coordinate the guluronate blocks of the alginate chains [25].

### 2.3. EPR Spectroscopy

The results obtained by IR spectroscopy and rheological measurements can provide information at a macroscopic or global level. These analyses are complemented by EPR measurements at nanoscale level, by analyzing the changes in the EPR parameters of the paramagnetic moieties covalently attached to the alginate chain or to alginate functionalized with adamantyl. In all experiments, the concentrations of salts and alginate were maintained at the same value to ensure the same cation/alginate ratio. The parameters evaluated from EPR spectroscopy were as follows: a_N_, which is usually sensitive to the polarity of the environment around paramagnetic moiety; and 2Azz, which is a parameter used to indirectly characterize the mobility of the paramagnetic moiety in the case of slow-motion dynamics but also when the polarity changes. The immobilization degree could be correlated with the 2Azz value. The higher the 2Azz value, the higher the probability that the rotation could occur around the a_z_ axis, and thus, a restricted motion is usually noticed.

The TEMPO concentration in the studied samples was determined by comparison with a TEMPO/water reference solution of a known concentration (5 × 10^−4^ M) measured under identical EPR conditions. The concentration was estimated from the ratio of the integrated EPR signal intensities (double integral), since the double-integrated signal was proportional with the spin concentration. After performing the necessary calculations, we obtained *I*_sample_/*I*_solution_ around 0.96. Therefore, the TEMPO concentration in the samples was slightly lower than 5 × 10^−4^ M. At this concentration, we did not observe any line broadening or spectral distortions indicative of significant dipole–dipole interactions; thus, dipole–dipole contributions were expected to be negligible under the studied conditions.

**Figure 5:**
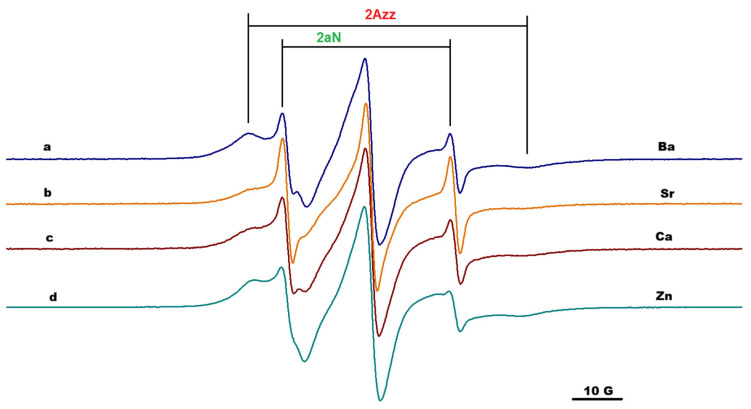
The EPR spectra of gels formed by complexation of Alg-AT in the presence of: (a) Ba^2+^, (b) Sr^2+^, (c) Ca^2+^, and (d) Zn^2+^.

From the previous study focused on the effect of host–guest interactions in functionalized alginate gels, we know that the parameters of the paramagnetic moieties attached to the chains in solutions of alginate functionalized with adamantyl or β-CD units indicated a slightly anisotropic motion, but remained in the fast regime dynamic, and the host–guest interactions in solutions had negligible effect [22]. Alginate gel formation in the presence of divalent cations induced changes in the EPR spectra of TEMPO moieties covalently attached to the alginate gels. In all cases, the EPR spectra showed a two-component feature: one corresponding to a slow dynamic that was almost immobilized, and the other one with a faster dynamic, similar with the spectrum of spin-labeled alginate in solutions (Figure 5). The slow component can be easily characterized by the distance between the outer peaks of the spectrum, 2Azz, while the fast component by 2a_N_, as is indicated in Figure 5 (spectrum *a*).

Due to the specific arrangement of G- and M-units from the alginate structure, divalent metal ions have different coordination preferences based on their ionic size. For example, the cross-linking between alginate and Ca^2+^ ions is possible through binding of the ion to pairs of guluronate units [26]. The component with restricted motion was associated with TEMPO moieties attached to G-blocks in the proximity of carboxylic groups involved in complexation with divalent cations, while the component characterized by faster motion was associated with the TEMPO units attached to the M-block or MG-block which were not involved in the interactions with divalent cations [23].

**Figure 6:**
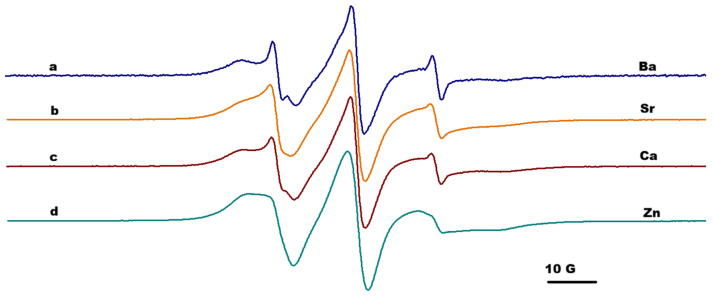
The EPR spectra of gels formed by complexation of Alg-Ad-AT in the presence of: (a) Ba^2+^, (b) Sr^2+^, (c) Ca^2+^, and (d) Zn^2+^.

**Figure 7:**
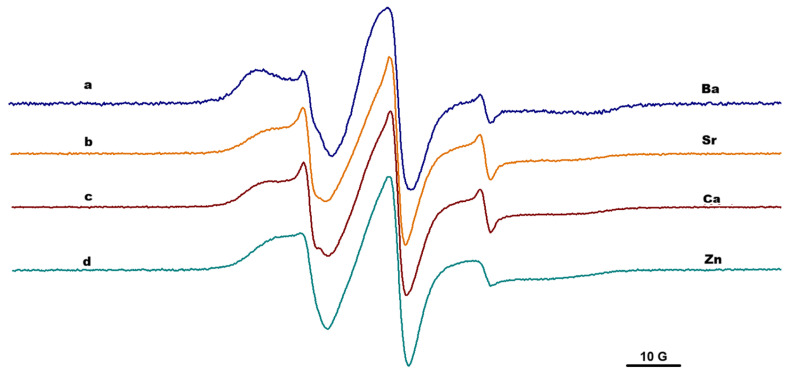
The EPR spectra of gels formed by complexation of Alg-Ad-AT and Alg-1,3-β-CD in the presence of: (a) Ba^2+^, (b) Sr^2+^, (c) Ca^2+^, and (d) Zn^2+^.

Figure 5, Figure 6 and Figure 7 present the EPR spectra of gels that were the result of complexation of Alg-Ad-TEMPO, Alg-Ad-TEMPO, and a mixture of Alg-1,3-β-CD/Alg-Ad-TEMPO with the four cations considered in this study. The EPR spectra correspond to the alginate gels (1%) formed in the presence of Zn^2+^, Ba^2+^, Sr^2+^ and Ca^2+^; and the gels were prepared using 1 M concentration of salts.

The values of effective 2Azz for the gels formed in the presence of Zn^2+^, Ca^2+^, Sr^2+^, and Ba^2+^ ions are shown in Table 2. The values of 2Azz varied in the following order: Ba > Ca > Sr > Zn. These values were irrespective of the gels generated in the presence of cations, and they correlated with the strength of the gels, e.g., Ba^2+^ formed a stronger alginate gel than Ca^2+^, as demonstrated by the rheology data. Divalent ions had a certain influence regarding the formation of the cross-linking network structure of the alginate; thus, the obtained gels showed different paramagnetic properties. The Ca^2+^ and Sr^2+^ had similar 2Azz values, most likely due to the close ionic radius [27].

The host–guest interactions established between the adamantyl groups and the cyclodextrin units attached to the alginate chains determined a more restricted motion of the paramagnetic groups (which is reflected in higher 2Azz values). At the same time, in the case of gels formed by adding cations to the solution consisting of Alg-Ad-AT and Alg-1,3-β-CD, a complexation of TEMPO units by CD units was also possible. Indeed, the simulation of the EPR spectra corresponding to the AlgAd-AT/Alg-1,3-β-CD gel led to lower a_N_ values corresponding to the free component, with the exception of Ca^2+^ (Table S2 from Supplementary Materials). The nitroxide radicals with τ less than 4 ns usually exhibit a liquid-type spectrum and are considered mobile/fast, while the nitroxide radicals with a correlation rotational time τ more than 4 ns exhibit a solid-type spectrum and are considered immobile/slow; likewise, polymers often exhibit distributions of correlation times, and thus, both fast and slow components may be observed from the spectra [28]. The EPR spectra of spin-labeled alginate gels formed in the presence of divalent cations showed this two-component feature: one mobile that was associated with TEMPO moieties attached to the alginate chain part that was not involved in complexation, and the other immobilized one, which was associated with TEMPO moieties found in proximity of the complexation sites. In general, the ratio between the above-mentioned components did not significantly change, irrespective of the ions used.

## 3. Conclusions

Altogether, the information collected from the experimental data by rheology, IR, and EPR measurements can prove that both the cation type and host–guest interactions between the appended groups to the alginate chains influenced the physical–chemical properties of the alginate gels. EPR spectroscopy is a sensitive method that could monitor the formation of cross-linking alginate gels, and, at the same time, gives a more profound understanding regarding the rotational motion of paramagnetic probes inside gels.

Rheology analysis can usually offer global information at a macroscopic scale regarding the behavior of dynamic polymer networks. Viscoelastic behavior was determined and shown to be significantly dependent on the polymeric system as well as the nature of the ions. The type of cross-linking ion significantly influenced the stiffness of hydrogels across all studied systems. Therefore, Ba^2+^ generally produced the most elastic and stable gel networks, followed by Ca^2+^ and Sr^2+^, whereas Zn^2+^ produced softer gels. The same pattern was also observed from EPR spectroscopy regarding the influence of divalent ions on the systems.

For the β-CD systems, the 2Azz values had the highest values, while the G′ moduli showed the lowest values across all the studied systems and ions. The local interactions studied via EPR showed a more restricted motion; therefore, we could conclude that a stronger local interaction influences the structure of gel.

When adamantyl was grafted on the backbone of alginate, we noticed that G′ moduli had the highest values while Azz recorded the lowest values, establishing that, in the presence of adamantyl, the spin probe sensed a less restricted motion. This behavior can be explained by the hydrophobic character of adamantyl, and at the same time by its bulkiness which made the adjacent complexation to be weaker.

On the other hand, the EPR measurements were performed to evaluate the anisotropic motion and the dynamic of the paramagnetic moiety in the presence of the divalent ions, offering more details about the local interactions. A more restricted motion of the paramagnetic groups was reflected in higher 2Azz values, which were also obtained when using Ba2+. However, for the other ions, there was a tendency to follow the same pattern as the rheological measurements. The formation of Ca^2+^ and Sr^2+^ alginate gels presented a tendency to have a less restricted motion compared to Ba^2+^, while Zn^2+^ was less restricted across all the studied systems.

## 4. Materials and Methods

### 4.1. Materials

Low-viscosity alginic acid sodium salt was purchased from Alfa Aesar (Karlsruhe, Germany). The 1-Adamantylamine, 4-amino-TEMPO, and calcium chloride from Sigma-Aldrich (St. Louis, MO, USA) were used. N-hydroxysulfosuccinimide (NHSS) was purchased from Fisher Scientific (Waltham, MA, USA), while 1-(3-dimethylaminopropyl)-3-ethylcarbodiimide hydrochloride (EDC) was purchased from Tokyo Chemical Industry Co., Ltd. (Tokyo, Japan). Zinc nitrate, strontium nitrate, and barium chloride were purchased from Merck (Darmstadt, Germany). β-cyclodextrin-TOS-(1,3)-diaminopropane, noted as β-CD-NH-(CH_2_)_3_-NH_2_, was synthesized according to Refs. [29,30].

### 4.2. Synthesis of Functionalized Alginates

Synthesis of alginate functionalized with –TEMPO was described previously [23]. Briefly, a solution of 1% alginate in distilled water was prepared. In order to activate the carboxylic groups, EDC and NHSS were added to the alginate solutions. The prepared mixture was kept under continuous stirring for 30 min to allow the compounds to react. Afterwards, solutions of adamantyl and 4-amino-TEMPO were added, and the reaction was completed overnight to obtain the alginate functionalized with adamantyl and TEMPO moieties. Similarly, alginate was functionalized with β-cyclodextrin-NH-(CH_2_)_3_ units as described in paper [22]. To separate the functionalized alginate from the reaction mixture, acetone was added in excess. Also, the precipitate was washed to remove unreacted TEMPO, adamantyl, and cyclodextrin molecules, and the solid was dried under reduced pressure [22,23].

### 4.3. Preparation of Alginate Gels

For the preparation of hydrogels, stock solutions of 1% concentration of functionalized alginates were obtained. A solution obtained by mixing equal volumes of adamantyl-TEMPO (Alg-AdAm) and β-CD-(CH_2_)_3_-Alginate (Alg-β-CD) solutions was prepared. The alginate gels were prepared for these solutions by adding dropwise 1M solutions of the following salts prepared in distilled water: Zn(NO_3_)_2_, Sr(NO_3_)_2_, CaCl_2_, or BaCl_2_.

### 4.4. Infrared Spectroscopy

The infrared spectra were collected using a Thermo Scientific Nicolet iS10 instrument (Thermo Fisher Scientific Inc., Waltham, MA, USA). The hydrogels were dried prior to analysis in order to reduce the possibility that the water content could strongly influence the absorption bands.

### 4.5. Rheology

Kinexus Pro rheometer (NETZSCH-Gerätebau GmbH, Selb, Germany) was used to measure the rheological parameters of the polymer systems that were cross-linked with ions. To keep the temperature at 25 °C, a Julabo CF41 compact circulating thermostat (JULABO GmbH, Seelbach, Germany) was used. The geometry was a 40 mm parallel plate with a rough surface and a gap of 0.8 to 1.2 mm. The amplitude sweep experiments were performed at a frequency of 1 Hz, modifying the shear strain until the deformation event occurred. We conducted frequency sweep experiments using a constant shear strain within the linear viscoelastic (LVE) region, covering a frequency range from 0.1 to 20 Hz. Moreover, the effect of 0.1 s^−1^ on the shear viscosity measurements of cross-linked polymer systems in MgCl_2_ was also evaluated. The data were shown in a logarithmic format.

### 4.6. EPR Spectroscopy

For the EPR measurements, a spectrometer from JEOL, model FA 100 (JEOL Ltd., Tokyo, Japan), equipped with a cylindrical type resonator TE011 was used. The parameters were set as follows: the frequency modulation 100 kHz; microwave power 1 mW; time constant 0.1 s; sweep time 240 s; conversion time 0.234 s; sweep width 150 G; and the modulation amplitude of 1 G—this was chosen because in solutions of TEMPO the width of the lines was around 1 G. The measurements were performed at 20 °C. The simulation of the obtained spectra was done using a computational package specially designed for spectral simulation and analysis, the EasySpin toolbox from MATLAB R2012a, v 7.14 (The MathWorks, Inc., Natick, MA, USA) [31].

## Figures and Tables

**Table 1:** Storage (G′) and loss (G″) moduli of alginate-based hydrogels at 0.5% shear strain in the presence of different divalent ions.

System	Alg		Alg-β-CD		Alg-AdAm		Alg-β-CD-AdAm	
Ion	G′ (Pa)	G″ (Pa)	G′ (Pa)	G″ (Pa)	G′ (Pa)	G″ (Pa)	G′ (Pa)	G″ (Pa)
Ba^2+^	2727.0 ± 118.1	300.4 ± 12.9	548.2 ± 29.1	92.5 ± 5.3	3270.0 ± 163.3	627.3 ± 40.7	850.5 ± 54.4	120.8 ± 5.9
Ca^2+^	661.3 ± 33.7	122.9 ± 6.3	319.4 ± 14.6	67.9 ± 3.6	781.2 ± 47.6	174.4 ± 9.9	301.8 ± 17.5	64.0 ± 3.4
Sr^2+^	960.1 ± 46.3	213.4 ± 9.6	73.7 ± 3.8	17.0 ± 0.8	538.4 ± 30.7	118.1 ± 6.6	192.7 ± 9.8	43.5 ± 2.3
Zn^2+^	372.3 ± 16.1	213.3 ± 8.9	708.3 ± 42.7	223.8 ± 11.7	417.0 ± 24.2	128.5 ± 7.1	378.6 ± 19.8	131.0 ± 7.9

**Table 2:** 2Azz (G) values for spin-labeled alginate gels in the presence of cations.

System	Ion Type			
	Ba^2+^	Sr^2+^	Ca^2+^	Zn^2+^
Alg-AT	56.7	55.2	56.1	54.6
Alg-AdAm-AT	56.2	55.4	55.7	53.9
Alg-β-CD-AdAm-AT	65.3	58.6	60.0	56.0

## Data Availability

The data presented in this study are available on request from the corresponding author.

## Supplementary Materials

The following supporting information can be downloaded at: https://www.mdpi.com/article/10.3390/gels12030217/s1, Table S1. Infrared spectra assignments for the alginate gels; Table S2. The EPR parameters for free and immobilized species obtained by simulation of experimental spectra; Figure S1. EPR experimental (black solid line) vs. simulated spectra (red dotted line). References [22,32,33,34,35,36] are mentioned in Supplementary Materials.
