# Supplementary material for: The Influence of Cations and Host–Guest Interactions on Alginate Gels Properties
Source: Gels. 2026 Mar 6;12(3):217. doi: 10.3390/gels12030217 (PMC13025125; doi:10.3390/gels12030217)
Supplement: Supplementary file 1 [file gels-12-00217-s001.zip › gels-4153588-supplementary.pdf]

gels

Supplementary Materials

The Influence of Cations and Host–Guest Interactions on Alginate Gels Properties

Gabriela Ioniță [1], Carmen Mihaela Topală [2], Elena-Erika Antonia [1], Mihaela Lavinia Ciutu [1], Alexandru Gabriel Bucur [1], Nusa Elena Hristea [1], Rodica Baratoiu [1], Ludmila Aricov [1], Anca Ruxandra Leonties [1] and Carla-Cezarina Pădurețu [1,*]

[1] Institute of Physical Chemistry–Ilie Murgulescu, Romanian Academy, 202 Splaiul Independentei, 060021 Bucharest, Romania; ige@icf.ro (G.I.); antoniaerika09@gmail.com (E.-E.A.); laviniamihaela.ciutu@yahoo.com (M.L.C.); abucur@icf.ro (A.G.B.); enhristea04@yahoo.com (N.E.H.); rodicad2003@yahoo.com (R.B.); laricov@icf.ro (L.A.); aleonties@icf.ro (A.R.L.)

[2] Faculty of Sciences, Physical Education and Computer Science, The National University of Science and Technology Politehnica Bucharest, Pitesti University Centre, 1 Targu din Vale Street, 110040 Pitesti, Romania; carmen.topala@upb.ro

* Correspondence: cpaduretu@icf.ro

Table S1. Infrared spectra assignments for the alginate gels

Sample	Ba^{2+}	Ca^{2+}	Zn^{2+}	Sr^{2+}	Assignment	References
Alg-M^{2+}	3460<br>3307<br>3225	3346	3235	3374	ν (O-H)	[1, 2, 3, 4]
	1636<br>1600	1600	1613	1602	ν (COO^-) asymmetric vibrations	
	1402	1430	1395	1435	ν (COO^-) symmetric vibrations	
	/	/	1312	1338	ν (NO_3^-)	
Alg-AdAm-M^{2+}	1028 (small intensity)	1024	1026	1024	ν (C-O-C), ν (C-O)	[1, 2, 3, 4, 5]
	3450<br>3315<br>3230	3346	3231	3360	ν (O-H)	
	1635<br>1598	1600	1613	1597	ν (C=O) stretching vibrations, ν(COO^-) asymmetric vibrations	
	1363	1431	1395	1400	ν(COO^-) symmetric vibrations	
	/	/	1312	1338	ν (NO_3^-)	
Alg-β-CD-AdAm-M^{2+}	1027	1024	1027	1026	ν (C-O-C), ν (C-O)	[1, 4, 5, 6]
	3450<br>3306<br>3223	3346	3231	3382	ν (O-H)	
	1634<br>1598	1603	1613	1600	ν(COO^-) asymmetric vibrations	
	1367	1431	1390	1435	ν(COO^-) symmetric vibrations	

 https://doi.org/10.3390/gels12030217

Table S2. The EPR parameters for free and immobilized species obtained by simulation of experimental spectra

System	Free species						Immobilized species					
	ax	ay	az	a_N,G	τ ^−10, s	Proportion	ax	ay	az	a_N,G	τ ^−10,s	Proportion
Alg_AT_Ba^{2+}	17.7	17.5	15.7	17.0	1.9	8.0	12.2	4.7	34.0	17.0	61.9	92.0
Alg_AdAm_AT_Ba^{2+}	17.1	16.9	16.9	17.0	2.3	6.8	12.2	4.6	34.1	17.0	57.8	93.2
Alg_AdAm_ β-CD_AT_Ba^{2+}	16.3	17.0	17.5	16.9	2.6	8.8	11.5	2.6	37.2	17.1	55.7	91.2
Alg_AT_Ca^{2+}	19.1	18.3	14.3	17.2	1.2	4.7	12.2	4.1	33.9	16.7	50.4	95.3
Alg_AdAm_AT_Ca^{2+}	17.2	17.1	17.2	17.2	1.6	7.8	11.9	5.5	33.4	16.9	58.2	92.2
Alg_AdAm_ β-CD_AT_Ca^{2+}	17.9	20.1	13.6	17.2	1.0	3.4	12.2	3.1	35.5	16.9	51.9	93.6
AlgAT_Zn^{2+}	17.5	19.0	15.3	17.3	1.6	7.8	11.5	3.0	36.4	17.0	64.1	92.2
Alg_AdAm_AT_Zn^{2+}	16.8	17.3	17.1	17.1	2.3	9.4	12.1	4.9	33.6	16.9	53.7	90.6
Alg_AdAm_ β-CD_AT_Zn^{2+}	17.0	17.0	16.9	17.0	2.0	9.4	12.2	3.9	34.4	16.8	51.3	90.6
Alg_AT_Sr^{2+}	16.3	17.4	17.2	17.0	2.4	16.1	12.1	4.9	34.8	17.3	52.0	83.9
Alg_AdAm_AT_Sr^{2+}	17.3	16.3	17.7	17.1	2.0	19.9	12.3	5.5	32.7	16.9	52.6	80.1
Alg_AdAm_ β-CD_AT_Sr^{2+}	15.6	17.8	17.3	16.9	1.2	8.8	12.6	4.0	34.1	16.9	55.3	91.2

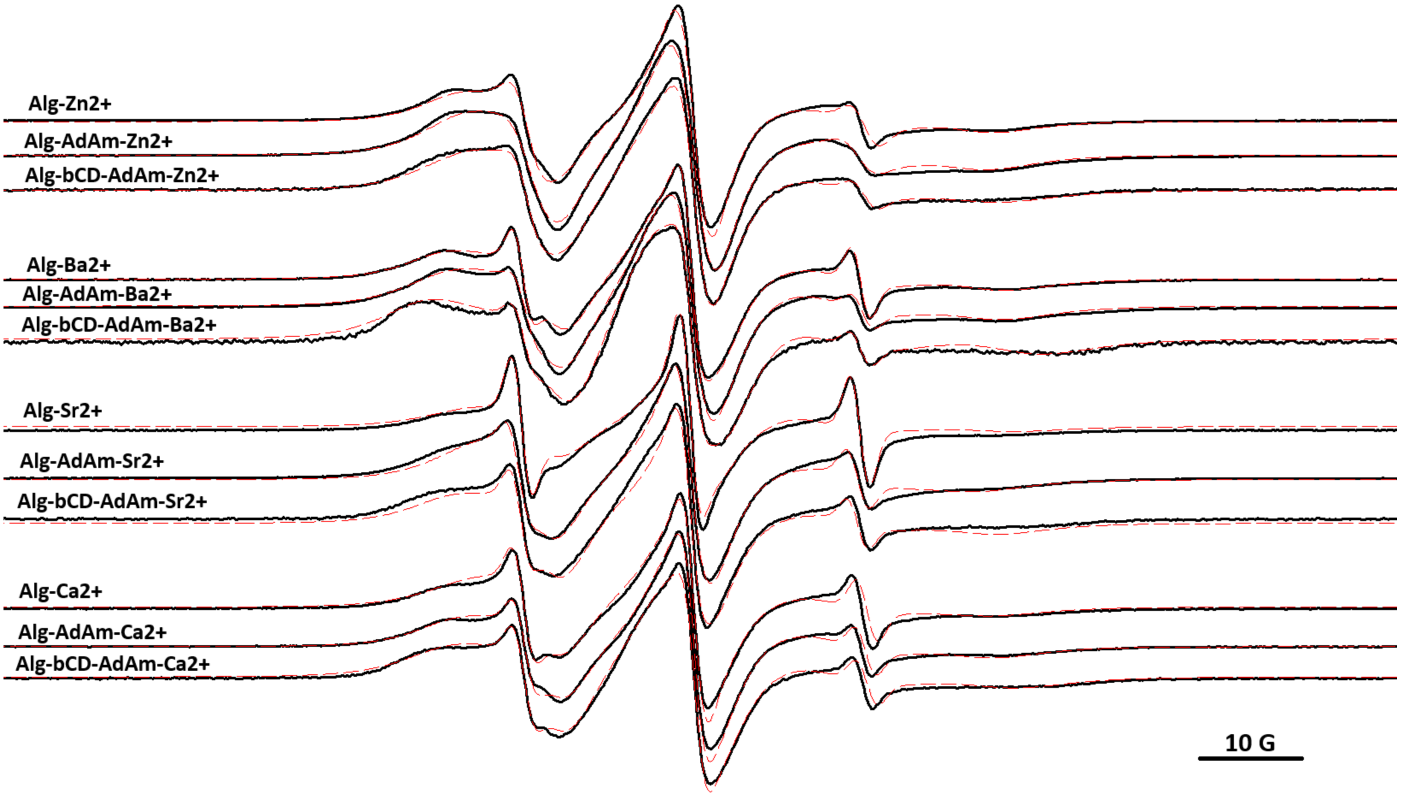

Fig. S1. EPR experimental (black solid line) vs. simulated spectra (red dotted line)

https://doi.org/10.3390/gels12030217

References

[1] Singh, B.; Chauhan, D. Barium Ions Crosslinked Alginate and Sterculia Gum-Based Gastroretentive Floating Drug Delivery System for Use in Peptic Ulcers. *Int. J. Polym. Mater. Polym. Biomater.***2011**, *60*, 684–705, https://doi.org/10.1080/00914037.2010.551354.

[2] Papageorgiou, S.K.; Kouvelos, E.P.; Favvas, E.P.; Sapalidis, A.A.; Romanos, G.E.; Katsaros, F.K. Metal–carboxylate interactions in metal–alginate complexes studied with FTIR spectroscopy. *Carbohydr. Res.***2010**, *345*, 469–473, https://doi.org/10.1016/j.carres.2009.12.010.

[3] Zhang, X.; Wang, L.; Weng, L.; Deng, B. Strontium ion substituted alginate-based hydrogel fibers and its coordination binding model. *J. Appl. Polym. Sci.***2019**, *137*, https://doi.org/10.1002/app.48571..

[4] Popescu, E.I.; Aricov, L.; Mocanu, S.; Matei, I.; Hristea, E.; Baratoiu, R.; Leonties, A.; Petcu, C.; Alexandrescu, E.; Ionita, G. Subtle influence on alginate gel properties through host–guest interactions between covalently appended cyclodextrin and adamantane units. *New J. Chem.***2021**, *45*, 8083–8091, https://doi.org/10.1039/d1nj01278a.

[5] Wang, A.; Jin, W.; Chen, E.; Zhou, J.; Zhou, L.; Wei, S. Drug delivery function of carboxymethyl-β-cyclodextrin modified upconversion nanoparticles for adamantine phthalocyanine and their NIR-triggered cancer treatment. *Dalton Trans.***2016**, *45*, 3853–3862, https://doi.org/10.1039/c5dt04900h.

[6] Pu, W.-F.; Yang, Y.; Wei, B.; Yuan, C.-D. Potential of a β-Cyclodextrin/Adamantane Modified Copolymer in Enhancing Oil Recovery through Host–Guest Interactions. *Ind. Eng. Chem. Res.***2016**, *55*, 8679–8689, https://doi.org/10.1021/acs.iecr.6b01793.
